# Gastric duplication cyst with ectopic pancreas in a teenager successfully resected by endoscopic submucosal dissection

**DOI:** 10.1186/s12893-022-01837-z

**Published:** 2022-11-07

**Authors:** Xiaodan Ye, Muqing Wang, Yuanyuan Wang, Daiying Lin, Xiaozhong Wang

**Affiliations:** 1 Department of Endoscopy Center, Shantou Central Hospital, 114 Waima Road, Shantou, 515041 Guangdong China; 2 Department of Pathology, Shantou Central Hospital, Shantou, China; 3 Department of Imaging, Shantou Central Hospital, Shantou, China

**Keywords:** Gastric duplication, Ectopic pancreas, Endoscopic submucosal dissection, Endoscopic ultrasonography, Case report

## Abstract

**Background:**

Gastric duplication cyst associated with ectopic pancreas is rare and we aimed to alert clinician to this congenital anomaly.

**Case presentation:**

A 15-year-old girl presented with intermittent vomiting. Gastroscopy showed a submucosal tumor with an approximate diameter of 40 mm in the anterior wall of the gastric antrum. The lesion had a central umbilication and was diagnosed preliminarily as gastric ectopic pancreas with pseudocyst formation on the basis of its appearance. However, computed tomographic scan showed a thick-walled cystic lesion with an enhanced outline of the cystic wall in the antrum of stomach, suggestive of duplication cyst. Serum amylase was normal. Endoscopic ultrasonography revealed a solid-cystic lesion; the solid portion were inhomogeneously mixed with echoes, and had indistinct border to muscularis propria; the cystic portion had echogenic internal mucosal layer and distinct border to muscularis propria. Endoscopic submucosal dissection (ESD) was suggested for the patient to relieve symptoms and diagnose the lesion definitely. The operation procedure was uneventful and the solid-cystic lesion was resected completely. Histopathologic examination revealed that the solid portion was ectopic pancreas, and the cystic portion was gastric duplication cyst. After resection, the patient discharged successfully and neither symptoms nor tumors recurred during the 9 months follow-up period.

**Conclusions:**

This is the first case of a solid-cystic lesion with central umbilication in the stomach diagnosed as gastric duplication cyst associated with ectopic pancreas. ESD could be an optional treatment to provide a definitive diagnosis.

**Supplementary Information:**

The online version contains supplementary material available at 10.1186/s12893-022-01837-z.

## Background

Gastric duplication cyst associated with ectopic pancreas is a rare congenital anomaly [1]. When present, symptoms can be due to the cyst itself or to the ectopic tissue. We have described here a case of gastric duplication cyst along with ectopic pancreas, mimicking ectopic pancreas with pseudocyst formation, resected by endoscopic submucosal dissection (ESD) finally. The aim of the study is to alert practitioners to this duplicate anomaly and recommend appropriate diagnostics and treatment.

## Case presentation

**Fig. 1:**
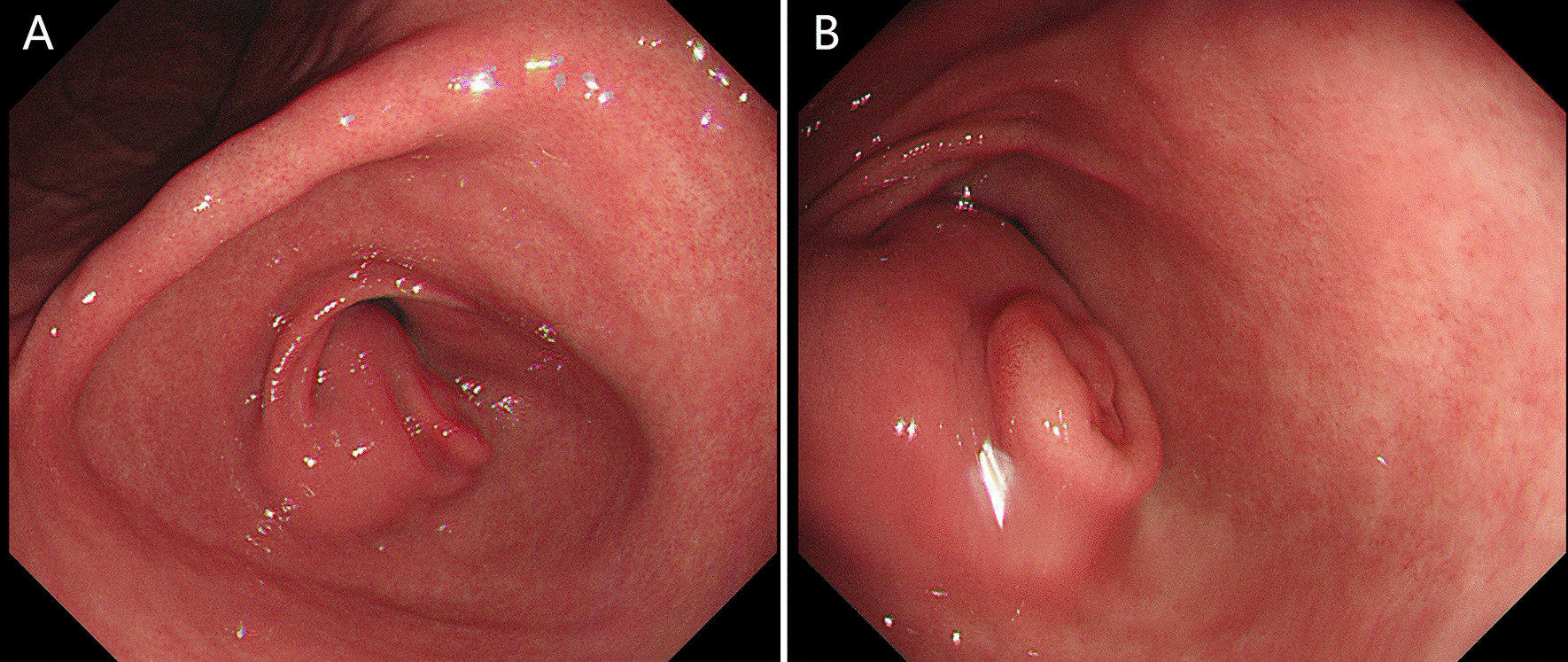
Gastroscopy showed a submucosal tumor with a central umbilication in the anterior wall of the gastric antrum. **A** Distant view. **B** Close-up view

**Fig. 2:**
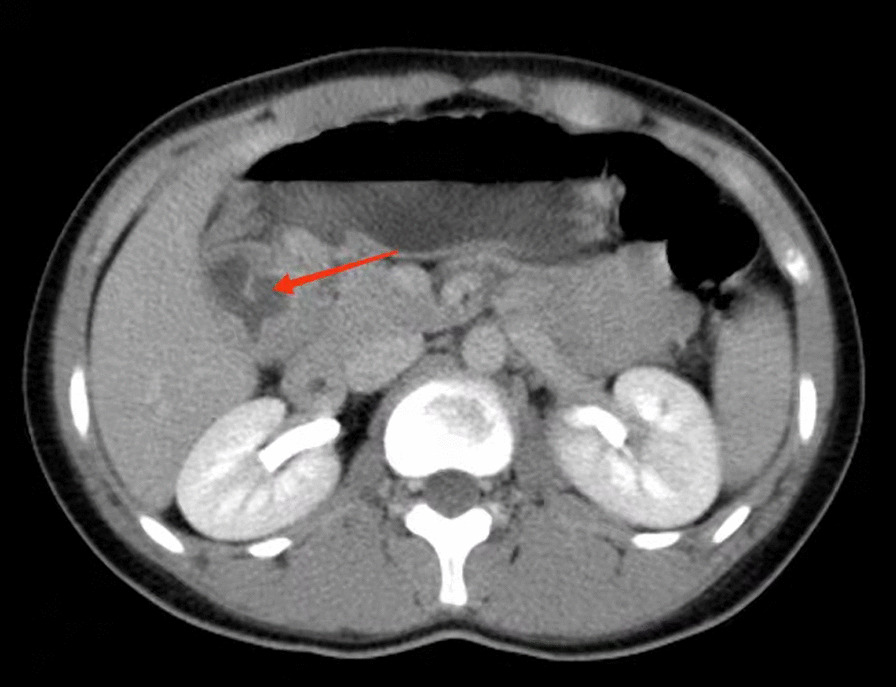
CT scan showed a thick-walled cystic lesion with an enhanced outline of the cystic wall in the antrum of stomach, suggestive of duplication cyst (arrow)

**Fig. 3:**
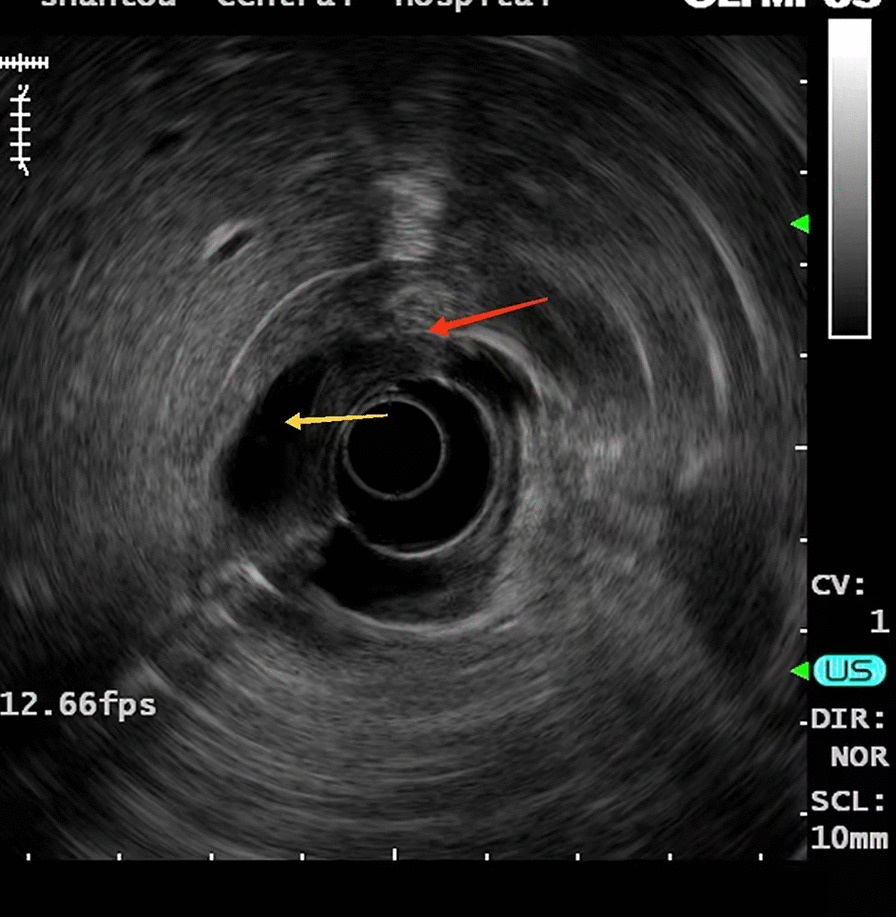
EUS revealed a solid-cystic lesion. The solid portion with mixed echogenicity, heterogeneity, and indistinct border origining from muscularis propria was observed (red arrow). The cystic portion demonstrated echogenic internal mucosal layer and distinct border to muscularis propria (yellow arrow)

**Fig. 4:**
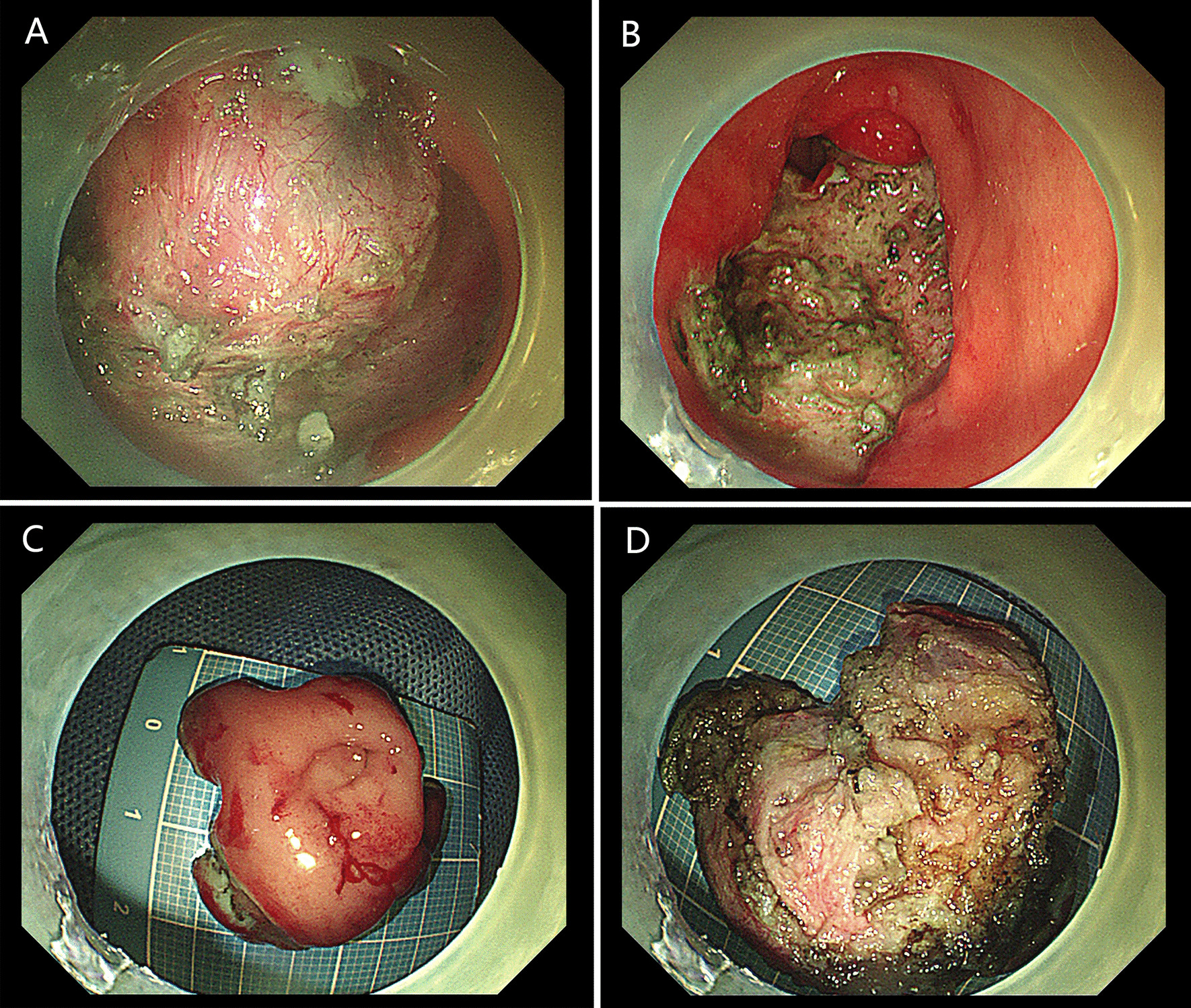
ESD procedure was uneventful and the solid-cystic lesion was resected completely. **A** Secured a clear view during dissection. **B** APC treated the wound. **C** Outer surface of the resected specimen. **D** Inner surface of the resected specimen

**Fig. 5:**
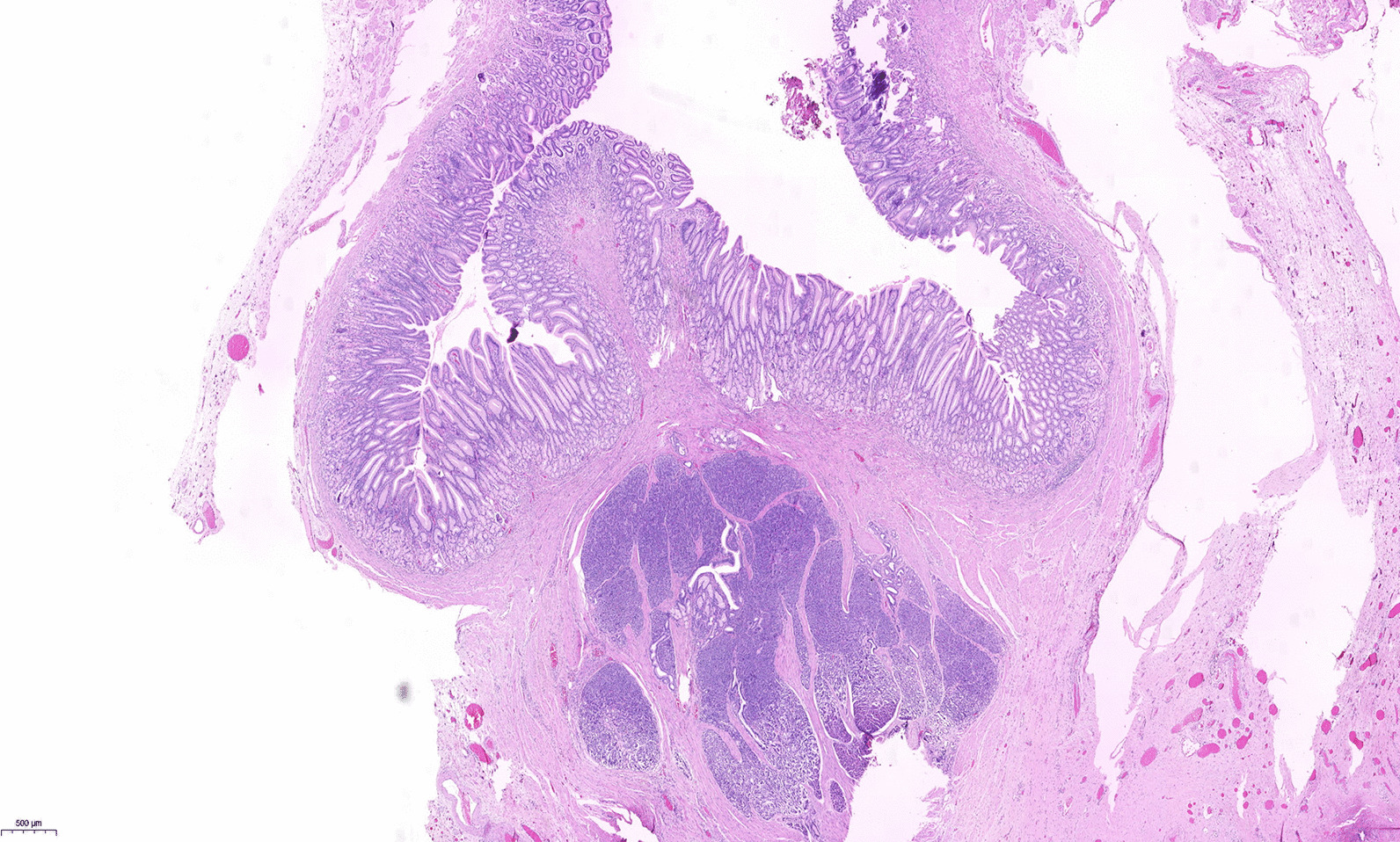
Gastric duplication cyst (Additional file 1: Fig S1) associated with ectopic pancreas (Additional file 2: Fig S2) was confirmed histopathologically (hematoxylin and eosin [H&E] stain, ×2). The equipment and software used to capture the images were the PANNORAMIC SCAN II (3D HISTECH, Hungary) and the CaseViewer, respectively

A 15-year-old girl presented with intermittent vomiting for the previous 5 years and worsened for 1 month. Medical history, physical examination, routine laboratory examination, electrocardiogram and chest X-ray were unremarkable. Gastroscopy showed a submucosal tumor with an approximate diameter of 40 mm in the anterior wall of the gastric antrum (Fig. 1). The lesion had a central umbilication and was diagnosed preliminarily as gastric ectopic pancreas with pseudocyst formation on the basis of its appearance. However, computed tomographic (CT) scan showed a thick-walled cystic lesion with an enhanced outline of the cystic wall in the antrum of stomach, suggestive of duplication cyst (Fig. 2). Serum amylase was 109.0 U/L (reference range: 15–150 U/L). Endoscopic ultrasonography (EUS) revealed a solid-cystic lesion with a diameter of 38 mm (Fig. 3). The solid portion with mixed echogenicity, heterogeneity, and indistinct border origining from muscularis propria was observed. The cystic portion demonstrated echogenic internal mucosal layer and distinct border to muscularis propria. There was no blood flow in the lumen. Subsequently, ESD was suggested for the patient to relieve symptoms and further clarify the lesion. The operation procedure was uneventful and the solid-cystic lesion was resected completely (Fig. 4). Histopathologic examination revealed that the solid portion was pancreatic tissues composed of acini, ducts, and islets of Langerhans; and the cystic portion’s cyst wall was lined by epithelium of gastric and surrounded by smooth muscle (Fig. 5). The horizontal/vertical margin was histologically free. As a result, gastric duplication cyst associated with ectopic pancreas was confirmed histopathologically. After resection, the patient discharged successfully and neither symptoms nor tumors recurred during the 9 months follow-up period.

## Discussion and conclusions

Gastric duplications represent about 7% of gastrointestinal tract duplications and are even more uncommon associated with ectopic pancreas [1]. The etiology is currently unknown, and most scholars believe that it is related to the abnormal development of gastrula [2]. The clinical presentations are usually asymptomatic or nonspecific symptoms such as abdominal pain, nausea, and vomiting. The nonspecific symptoms may have resulted from overdistention of the cyst, peptic ulcer formation, or rupture with peritonitis. Gastric duplications usually presented with symptoms in childhood, with less than 25% being detected after the age of 12 [3]. In this study, we present our experience in the diagnostics and treatment of gastric duplication cyst associated with ectopic pancreas in a teenager. To the best of our knowledge, this is the first case of gastric duplication cyst associated with ectopic pancreas with the characteristics of umbilication, which is easy to be mistaken for ectopic pancreas with pseudocyst formation endoscopically. Though ectopic pancreatic tissue presented in 37% of gastric duplication cysts, the size of ectopic pancreas reported in previous literatures is generally microscopic [4]. Zhou et al. [5] demonstrated that the three common characteristics to help identify gastric ectopic pancreas including the site in the gastric antrum, central umbilication, and the complete mucous membrane. Ectopic pancreas may lead to pancreatitis with pseudocyst, and can present as a cystic mass [6].

Preoperative diagnosis of gastric duplication cyst with ectopic pancreas is difficult, largely due to their rarity and the absence of characteristic findings. CT and EUS may provide some informative findings. Contrast-enhanced CT scan typically demonstrates gastric duplication cyst as a thick-walled cystic lesion with enhancement of the inner lining [3]. However, CT seems to be not very helpful in the diagnosis of gastric ectopic pancreas [5]. EUS could provide useful information regarding tumor size, layer of origin and echogenicity. Passos et al. [3] revealed EUS features of gastric duplication cyst that a cyst with an echogenic internal mucosal layer and a hypoechoic intermediate muscular layer. Liu et al. [7] demonstrated that gastric ectopic pancreas generally appears as a heterogeneous hypoechoic mass with poorly defined borders originating from the deep mucosa to muscularis propria layer. On the basis of EUS findings, definitive diagnosis was achieved in a few cases. Catalano et al. [8] showed that ESD offers definitive treatment of submucosal tumor (SMT) in cases in which the available endoscopic techniques such as EUS do not provide a definitive diagnosis. ESD is a minimal invasive technology and could allow en bloc resection for gastric subepithelial lesions, even those originated from muscularis propria [5]. Complete resection is the key to the treatment and prevention of recurrence. In our case, EUS revealed the solid portion of the lesion with an indistinct border to muscularis propria. During dissection, the solid portion was closely adhered to the muscularis propria; therefore, a clip attached with dental floss was applied to fully expose the lesion, thereby securing a clear view and reducing the occurrence of perforations (Fig. 4A). Kim et al. [9] noted similar findings and suggested that dental floss and clip traction is an efficient and safe method for en bloc resection. Besides, argon plasma coagulation (APC) was used to treat the wound, so as to avoid residue, and stanch bleeding (Fig. 4B). Noh et al. [10] indicated that the cauterization effect at the deep resection margin of the lesion during ESD generally ablates any remnant cells. Compared with ESD, conventional treatment of open or laparoscopic surgery is overly invasive. In addition, some scholars recommended regular follow-up for asymptomatic lesions [2].

In conclution, gastric duplication cyst with ectopic pancreas should be included in the differential diagnosis of a solid-cystic lesion with central umbilication in the stomach. ESD could be a minimal invasive treatment to provide a definitive diagnosis.

## Supplementary Information

**Additional file 1: Fig S1.** The cystic portion’s cyst wall was lined by epithelium of gastric and surrounded by smooth muscle (hematoxylin and eosin [H&E] stain, ×10).

**Additional file 2: Fig S2.** The solid portion was pancreatic tissues composed of acini, ducts, and islets of Langerhans (hematoxylin and eosin [H&E] stain, ×10).

## Data Availability

The datasets used in the current study are available from the corresponding authors on reasonable request.

## Abbreviations

ESD: Endoscopic submucosal dissection
CT: Computed tomographic
EUS: Endoscopic ultrasonography
SMT: Submucosal tumor
APC: Argon plasma coagulation
