# Supplementary figures and images for: Gastric duplication cyst with ectopic pancreas in a teenager successfully resected by endoscopic submucosal dissection
Source: BMC Surg. 2022 Nov 7;22:381. doi: 10.1186/s12893-022-01837-z (PMC9639261; doi:10.1186/s12893-022-01837-z)

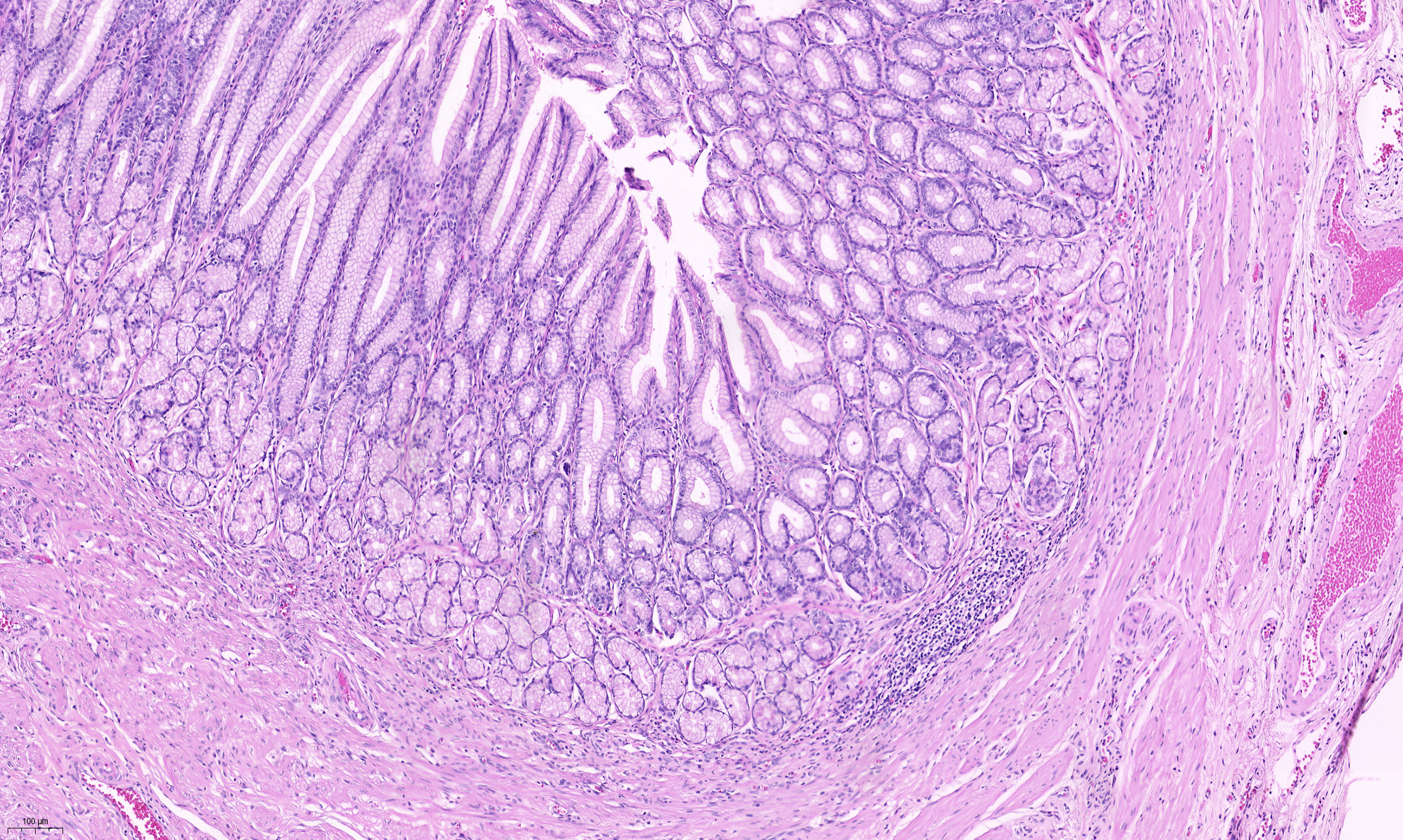

Supplement: Supplementary file 1 — Additional file 1: Fig S1. The cystic portion’s cyst wall was lined by epithelium of gastric and surrounded by smooth muscle (hematoxylin and eosin [H&E] stain, ×10). [file 12893_2022_1837_MOESM1_ESM.tif]

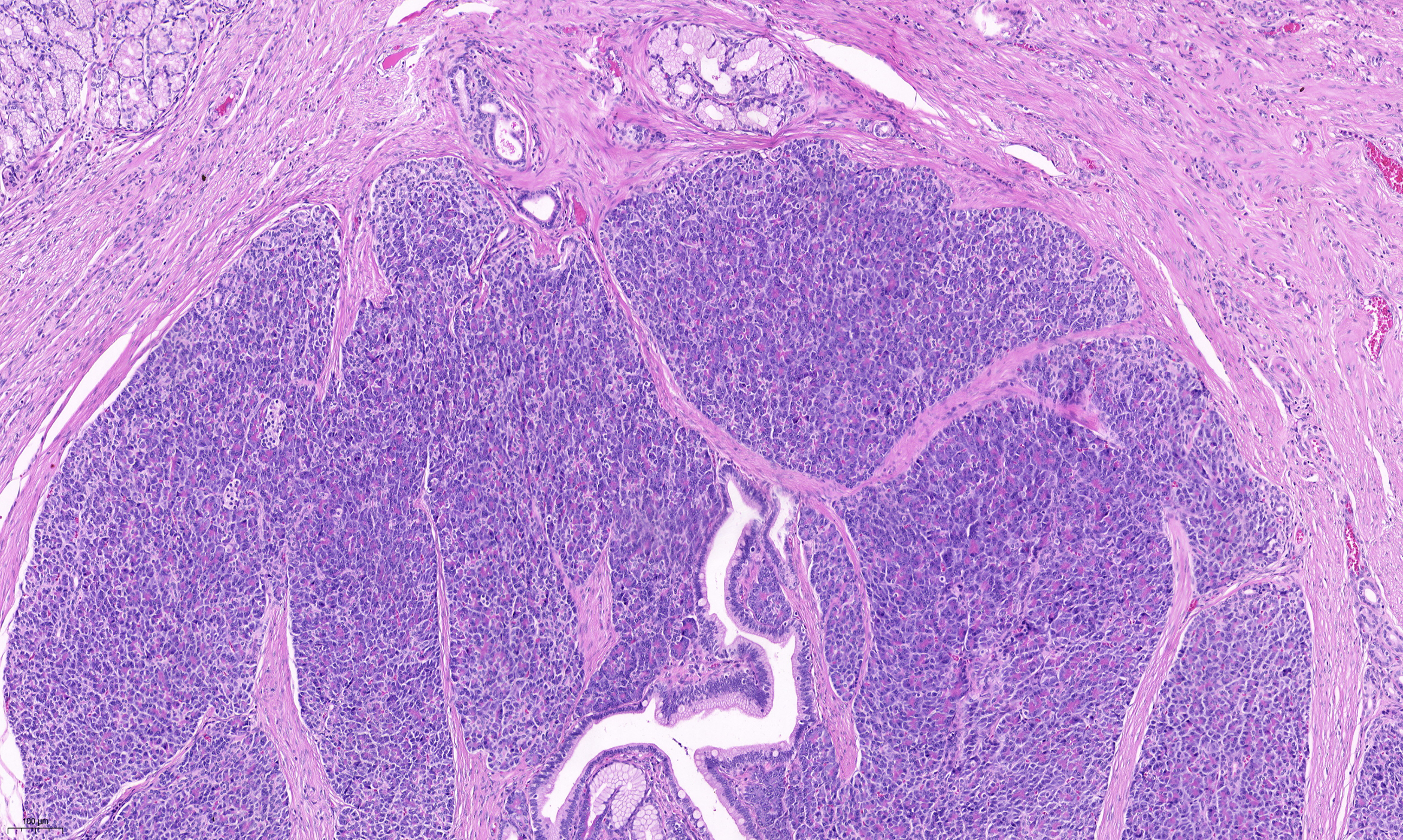

Supplement: Supplementary file 2 — Additional file 2: Fig S2. The solid portion was pancreatic tissues composed of acini, ducts, and islets of Langerhans (hematoxylin and eosin [H&E] stain, ×10). [file 12893_2022_1837_MOESM2_ESM.tif]
